# Réactualisation de la limite nord de distribution des glossines au Tchad dans un contexte de changement global

**DOI:** 10.48327/mtsi.v4i1.2024.392

**Published:** 2024-02-29

**Authors:** Brahim GUIHINI MOLLO, Moukhtar ALDJIBERT, Juste DARNAS, Wilfrid YONI, Lassina SANOGO, Issiaka BARRY, Djouk SIGNABOUBO, Ramadan KALKI, Djaklessam HAIWANG, Sylvain BIÉLER, Arada Izzedine ABDEL AZIZ, Giuliano CECCHI, Fabrice COURTIN, Philippe SOLANO

**Affiliations:** 1 Institut de recherche en élevage pour le développement (IRED), Ndjaména, Tchad; 2 Programme national de lutte contre la trypanosomiase humaine africaine (PNLTHA), Moundou, Tchad; 3 Centre international de recherche-développement sur lélevage en zone subhumide (CIRDES), Bobo-Dioulasso, Burkina Faso; 4 Université de N'Djaména, Tchad; 5 Foundation for innovative new diagnostics (FIND), Genève, Suisse; 6 Organisation des Nations unies pour l'alimentation et l'agriculture (FAO), Division de la production et de la santé animales, Rome, Italie; 7 Institut de recherche pour le développement (IRD), UMR Intertryp IRD-CIRAD, Représentation IRD à Ouagadougou, Burkina Faso; 8 Institut de recherche pour le développement (IRD), UMR Intertryp IRD-CIRAD, Université de Montpellier, France

**Keywords:** Tsé-tsé, Limite nord, Distribution, Trypanosomoses, Changement global, Mayo-Kebbi, Moyen-Chari, Salamat, Mandoul, Logone Oriental, Logone Occidental, Tandjilé, Guéra, Chari-Baguirmi, Tchad, Afrique subsaharienne, Tsetse, Northern distribution limit, Trypanosomoses, Global change, Mayo-Kebbi, Moyen-Chari, Salamat, Mandoul, Logone Oriental, Logone Occidental, Tandjile, Guera, Chari-Baguirmi, Chad, Sub-Saharan Africa

## Abstract

**Introduction - Justification:**

Les glossines (ou mouches tsé-tsé) sont responsables de la transmission des trypanosomes aux humains et aux animaux. Au Tchad, seule la partie méridionale du pays possède des conditions écologiques favorables à la présence des glossines, dont la dernière cartographie date de 1996. Ce travail a visé à actualiser la distribution des glossines au Tchad, en particulier sa limite nord, dans un contexte de changement global, pour aider les programmes de contrôle des trypanosomoses africaines.

**Matériels et méthodes - Résultats:**

Au total, sur les 217 pièges biconiques posés en 2021 et 2022 d'ouest en est, 1 024 glossines de 3 taxons différents ont été capturées, parmi lesquelles *Glossina morsitans submorsitans* (57 %), *G. tachinoides* (39 %) et *G. fuscipes fuscipes* (4 %). Au-delà des distributions de chaque espèce, cette étude met en évidence un fort recul vers le sud de la limite nord de distribution en comparaison avec les études antérieures de 1966 et 1996. Nous observons également une fragmentation croissante de la présence des tsé-tsé, en « poches » discontinues.

**Discussion - Conclusion:**

Le recul vers le sud de la limite nord de distribution des tsétsé dans le sud du Tchad traduit les effets combinés du changement climatique et de l'augmentation de la pression humaine sur les terroirs. Certains foyers historiques de trypanosomiase humaine africaine sont dorénavant hors de la limite de présence des tsé-tsé, tandis que d'autres doivent continuer de faire l'objet d'une surveillance. Ces résultats vont aider les programmes nationaux à atteindre les objectifs de contrôle et d’élimination des trypanosomoses humaines et animales.

## Abstract (en)

**Background - Rationale:**

Tsetse flies *(Diptera: Glossinidae)* are obligate bloodfeeders that occur exclusively in Sub-Saharan Africa, where they are the vectors of trypanosomes causing HAT (human African trypanosomiasis) and AAT (African animal trypanosomiasis). In Chad, tsetse flies occur only in the most southern part of the country because of its favorable bioclimatic conditions. However, despite the importance of HAT and AAT in this country, very little is known about the current tsetse distribution, in particular its northern limit, which is of key importance for the surveillance of these diseases.

**Material and methods - Results:**

A total of 217 biconical traps were deployed in 2021 and 2022 from the West to the East around the formerly known northern limit, resulting in 1,024 tsetse caught belonging to three different taxa: *Glossina morsitans submorsitans* (57%), *G. tachinoides* (39%) and *G. fuscipes fuscipes* (4%). In addition to the information gathered on the presence/absence of each tsetse taxon, we show a strong North-South shift of the northen tsetse distribution limit as compared to the previous works from 1966 to 1996, and a growing spatial fragmentation in more and more discrete pockets of tsetse presence.

**Discussion - Conclusion:**

This North-South shift of the northern tsetse distribution limit in Chad is the likely consequence of the combined effect of severe draughts that affected the country, and increasing human pressure on land. This update of the tsetse northern limit will be of help to the national programmes in charge of HAT and AAT.

## Introduction

Les glossines, ou mouches tsé-tsé, sont des vecteurs biologiques des trypanosomoses africaines. Ces insectes hématophages dans les deux sexes sont répartis exclusivement en Afrique subsaharienne en différents groupes taxonomiques selon leurs préférences d'habitats [17, 32]. Les glossines sont responsables de la transmission des trypanosomes aux humains et aux animaux, qui peuvent contracter par la suite respectivement la trypanosomiase humaine africaine (THA ou maladie du sommeil), ou la trypanosomose animale africaine (TAA), connue sous le nom de « Nagana » [11, 22]. En Afrique centrale et occidentale, la THA est causée par *Trypanosoma brucei gambiense,* responsable de la forme chronique, tandis qu'en Afrique orientale et australe, elle est causée par *T. b. rhodesiense,* donnant lieu à la forme aiguë [3]. La TAA est causée par plusieurs espèces et sous-espèces de trypanosomes dont *T. congolense, T. b. brucei, T. vivax* et *T. simiae* sont les plus courants. Parmi ces trypanosomes, *T. congolense* et *T. vivax* sont considérés comme les plus importants en raison de leur virulence et de leur impact économique et social sur la production agricole et animale [1, 2, 4].

La mouche tsé-tsé est reconnue comme une contrainte majeure au développement de l'agriculture et de l’élevage dans 36 pays d'Afrique subsaharienne, de par son rôle de vecteur des trypanosomoses. La TAA réduit la production laitière de 10 à 40 %, le nombre de têtes de bétail de 10 à 50 % et la production végétale de 2 à 10 % [1]. En outre, les pertes directes et indirectes résultant de l'impact de la maladie sur l’économie africaine ont été estimées à 5 milliards d'euros par an [1]. Le contrôle de la TAA bénéficierait au secteur agricole pour un montant estimé à 1 300 millions de dollars par an [29]. Ainsi, la « campagne panafricaine pour l’éradication de la tsé-tsé et de la trypanosomose » (PATTEC) a été lancée il y a une vingtaine d'années par l'Union africaine pour surmonter les pertes économiques dues aux infections par les trypanosomes chez le bétail [28]. Plus récemment, le développement du Parcours de contrôle progressif (PCP) de la TAA par l'Organisation des Nations unies pour l'alimentation et l'agriculture (FAO) [11] visant à contrôler durablement, voire à éliminer les mouches tsé-tsé et les trypanosomoses, a souligné les besoins de compréhension de l’épidémiologie de la maladie dans différents socio-écosystèmes. Pour cette raison, plusieurs études ont été entreprises sur les infections trypanosomiennes chez les mammifères et les mouches tsé-tsé au Tchad, non seulement dans les foyers de maladie du sommeil, mais aussi dans les zones infectées à fort potentiel agricole [16,18,20,21,30,34].

Au Tchad, pays sahélien, seule la partie méridionale du pays offre aujourd'hui les conditions bioclimatiques permettant la présence de la mouche tsé-tsé. L’élevage y est considéré comme l'une des activités économiques les plus importantes avec plus de 110 millions de têtes de bovins, ovins, caprins, chameaux, chevaux et porcs [19]. La partie sud du Tchad se situe dans la zone soudanienne et dispose d'un très important potentiel agro-pastoral. Toutefois elle est fortement infestée par les glossines, ce qui se traduit par une prévalence d'infections trypanosomiennes allant jusqu’à 41,3 % chez les bovins [16]. Non seulement elle est la principale zone de production agricole du Tchad du fait des conditions bioclimatiques favorables, mais elle accueille également plusieurs milliers de bovins (transhumants, nomades) en saison sèche en raison de la disponibilité en eau et en pâturages. Parallèlement, les espaces protégés qui sont présents dans cette zone, abritent une grande diversité d'animaux sauvages pouvant constituer un réservoir pour la TAA et des hôtes nourriciers pour les tsétsé. Le sud du Tchad est également touché par la THA, avec 5 foyers historiques (Tapol, Moissala, Goré, Mandoul, Maro) [15, 16, 18, 31] dont les deux derniers sont encore actifs et font l'objet d'une lutte antivectorielle depuis plusieurs années [21]. En dehors de ces foyers, peu d'attention a été accordée ces dernières années aux zones susceptibles d'abriter des mouches tsé-tsé, telles que les rives des lacs et les zones tampons des espaces protégés, qui sont pourtant soumises à une pression agricole et un élevage extensif. Les dernières études d'envergure datent de plus de 25 ans [9, 14]. L’étude pionnière réalisée par Gruvel [14] a permis de connaître la distribution géographique des trois espèces et sous-espèces de glossines présentes, à savoir *G. morsitans submorsitans, G. fuscipes fuscipes* et *G. tachinoides.* Plus tard, Cuisance [9] a confirmé la présence de ces trois taxons mais notait déjà une réduction conséquente de leur aire de distribution confinée à l'extrême sud du pays. *G. morsitans submorsitans* et *G. tachinoides* sont considérés comme des vecteurs de trypanosomes causant les TAA, alors que *G. f. fuscipes* est le seul vecteur de THA au Tchad. Compte tenu des processus d’élimination de la THA [13] et du PCP de la TAA [11] en cours à l’échelle continentale, il apparaissait essentiel d'actualiser les données sur la distribution des glossines. Ceci afin de contribuer à l’évaluation du risque trypanosomien et d'aider les programmes nationaux dans leur stratégie et mise en œuvre de contrôle des trypanosomoses africaines au Tchad. Pour ce faire, le présent travail s'est focalisé sur la mise à jour de la limite nord de la répartition des tsé-tsé. Ces enquêtes constituent la première étape vers l’élaboration d'un atlas national des mouches tsé-tsé pour le Tchad, à l'exemple de ce qui a commencé à être réalisé dans d'autres pays, par exemple au Burkina Faso [24].

## Méthodologie

Les données de la présente étude sont comparées à celles des deux travaux précédemment cités [9, 14].

### Zone d’étude

**Figure 1:**
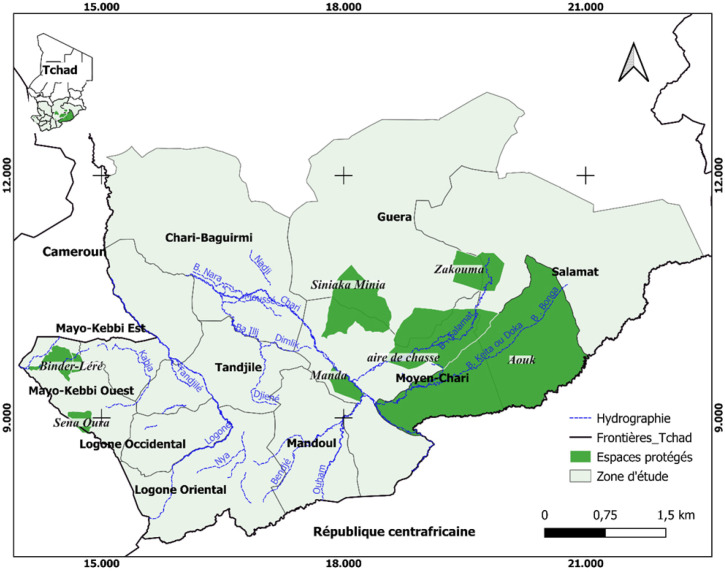
Carte de la zone d’étude située à l'extrême sud du Tchad, seule partie affectée par la présence de mouches tsé-tsé. Y figurent notamment les noms des régions, des principaux cours d'eau, et des zones protégées abritant potentiellement de la faune sauvage Map of the study area showing the southernmost part of Chad, which is the only part of the country where tsetse still occur. The names of regions, main rivers, national parks and wildlife protected areas are shown

Les activités de terrain ont été menées dans le sud et le sud-est du Tchad et englobent les 9 provinces suivantes : Mayo-Kebbi, Moyen-Chari, Salamat, Mandoul, Logone Oriental, Logone Occidental, Tandjilé, Guéra et Chari-Baguirmi (Fig. 1). Ces provinces sont situées dans les bassins hydrographiques du Chari et du Logone. Dans cette vaste région, la pluviométrie annuelle est de 800 à 1200 mm, répartie en deux saisons : une saison des pluies de mai à octobre et une saison sèche de novembre à avril. La température moyenne annuelle est de 27 °C, tandis que l'humidité relative moyenne annuelle est de 50 %. Les habitants de ces provinces pratiquent l’élevage, l'agriculture extensive, principalement céréalière, et la pêche. Cette partie du pays abrite 6 espaces protégés au sein desquels subsiste de la faune sauvage : le Parc national de Zakouma, la Réserve de Siniaka Minia, l'aire de chasse du Lac Iro, la Réserve de Manda et les zones de Binder Léré et Sena Oura. En plus des éleveurs sédentaires de bétail déjà présents en toutes saisons, ces provinces sont largement fréquentées par les éleveurs transhumants en saison sèche du fait de la disponibilité en eau et en pâturages.

### Identification des sites de piégeage des glossines

Cette étape a consisté à chercher des sources historiques sur la distribution des glossines au Tchad. Les principales sources utilisées ont été les publications de Gruvel, 1966 [14] et de Cuisance, 1996 [9], ainsi que des rapports entomologiques et épidémiologiques des institutions nationales IRED et PNLTHA. Parallèlement à ces données historiques, la structure du réseau hydrographique et l’état de conservation de la végétation riveraine ont été caractérisés via Google Earth. Ainsi, en croisant les lieux où des glossines avaient été capturées par le passé à l’état actuel de la végétation riveraine, plusieurs sites de capture ont été identifiés. Les coordonnées géographiques de ces sites ont été partagées avec l’équipe de terrain, qui les a introduites dans leurs GPS. À ces sites, ont été ajoutés des sites de la province du Chari-Baguirmi, compte tenu d'informations reçues mentionnant la présence de glossines. Lors des déplacements de l’équipe sur le terrain, des gîtes apparaissant comme particulièrement favorables aux glossines ont également bénéficié de la pose de pièges.

### Protocole de piégeage des glossines

En mars et avril 2021, puis en avril 2022 pour compléter la répartition géographique des pièges posés à la suite des évènements survenus au Tchad qui avaient interrompu la mission de 2021, un total de 217 pièges biconiques Challier-Laveissière [5] ont été posés, parmi lesquels 171 pièges positionnés sur et autour des sites préalablement prospectés par Gruvel et Cuisance, et 46 placés sur des lieux identifiés sur le terrain comme étant potentiellement favorables à la présence de glossines. Ces 217 pièges ont été déployés sur 86 sites répartis dans 69 villages de 18 départements. La distance entre pièges posés sur un même site était d'environ 100 mètres, variant en fonction de la végétation et de l'accessibilité. Chaque piège a été géo-référencé avec attribution d'un identifiant unique. La durée de piégeage a varié entre 2 h et 48 h en fonction de l'accessibilité du site et de la présence de glossines. À chaque visite, les cages contenant les glossines étaient collectées et conservées dans un conteneur au frais avant d’être transportées au camp de travail pour le dénombrement et l'identification des espèces. Les tsé-tsé capturées ont été identifiées par espèce à l'aide des clés morphologiques d'identification des glossines [12, 23]. Une fiche d'enquête entomologique a été utilisée pour enregistrer l'ensemble des résultats des piégeages (localisation administrative, coordonnées géographiques, numéro de piège posé, nombre de glossines capturées, espèces de glossines capturées).

### Analyse des données

Les données collectées sur le terrain (nombre de mouches, coordonnées GPS des pièges) ont été saisies sous Excel et transférées dans le logiciel QGIS pour la production des cartes.

## Résultats

### Distribution des glossines par région et par espèce

Sur les 217 pièges déployés, 58 ont capturé des glossines. Le nombre total de glossines capturées a été de 1 024, dont 45 *G. f. fuscipes* (4 %), 397 *G. tachinoides* (39 %) et 582 *G. morsitans submorsitans* (57 %) (Tableau I). Ainsi, 159 pièges n'ont capturé aucune glossine alors qu'ils étaient posés soit sur des sites où des glossines étaient présentes lors des enquêtes de Gruvel et Cuisance, soit sur des sites considérés comme potentiellement favorables.

**Tableau I:** Répartition des espèces de glossines par province prospectée Distribution of tsetse species in the prospected provinces

Province	Gff N (%)	Gt N (%)	Gms N (%)	G spp N (%)
Chari-Baguirmi	0	1 (0,25)	2 (0,34)	3 (0,29)
Guéra	1 (2,22)	1 (0,25)	61 (10,48)	63 (6,15)
Logone Oriental	35 (77,77)	0	0	35 (3,41)
Logone Occidental	0	0	0	0
Mandoul	0	0	0	0
Mayo-Kebbi Ouest	9 (20)	328 (82,62)	0	337 (32,9)
Moyen-Chari	0	30 (7,55)	71 (12,20)	101 (9,86)
Salamat	0	37 (9,31)	448 (76,9)	485 (47,36)
Tandjilé	0	0	0	0
**Total**	**45**	**397**	**582**	**1 024**

*Gff : Glossina fuscipes fuscipes; Gt : G. tachinoides; Gms : G. morsitans submorsitans; G spp : Glossina spp; N : effectif; % : pourcentage*

Sur les 9 provinces investiguées, des mouches tsé-tsé ont été capturées dans 6, alors que 3 n'en ont montré aucune.

*G. f. fuscipes* a été capturée dans 3 provinces (principalement dans le Logone Oriental avec 77 % des captures), *G. tachinoides* a été capturée dans 4 provinces (principalement dans le Mayo-Kebbi Ouest avec 82 % des captures) et *G. morsitans submorsitans* a été capturée dans 5 provinces (principalement dans le Salamat avec 47 % des captures).

**Figure 2:**
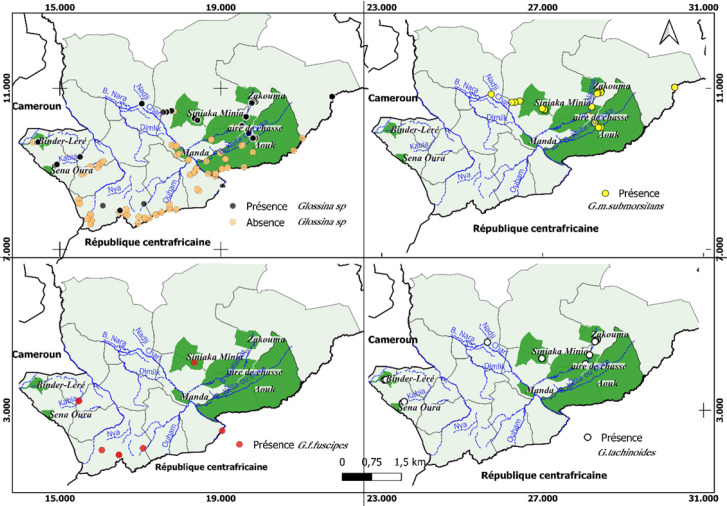
Distribution spatiale des glossines capturées. Chacune des 4 cartes montre respectivement : les lieux de capture et de non-capture de tsé-tsé toutes espèces confondues (en haut à gauche), les lieux de capture de *G. morsitans submorsitans* (en haut à droite), de *G. f. fuscipes* (en bas à gauche), et de *G. tachinoides* (en bas à droite) Spatial distribution of the tsetse caught. Each of the 4 maps shows respectively: the presence and absence sites of capture of tsetse, all species combined (top center), the capture sites of G. morsitans submorsitans (top right), of G. f. fuscipes (bottom center), and of G. tachinoides (bottom right)

*G. morsitans submorsitans* est présente exclusivement dans les parties nord et est de notre zone d’étude correspondant à la zone climatique des savanes boisées, au niveau du Parc national de Zakouma, de la réserve de Siniaka Minia et des aires de chasse du Lac Iro et de l'Aouk, situés dans les régions de Guéra, Salamat et Moyen-Chari où subsiste de la faune sauvage. *G. f. fuscipes* quant à elle, reste présente dans la zone sud à la frontière avec la République centrafricaine et le Cameroun, c'est-à-dire la partie la plus humide du pays ainsi qu'autour du Bahr Siniaka Minia (réserve de Siniaka Minia) et de la rivière Kabia. Elle est le seul vecteur présent dans les foyers de THA dans le Logone Oriental et le Mayo-Kebbi à l'est. *G. tachinoides* est présente dans la zone de savane à l'Est, généralement autour des cours d'eau permanents et temporaires, mais est totalement absente à l'extrême Sud. Elle est trouvée dans quelques endroits à faible densité dans la province de Mayo-Kebbi près de la frontière camerounaise (Fig. 2).

### Évolution de la limite nord des glossines au Tchad de 1966 à 2021-2022

**Figure 3:**
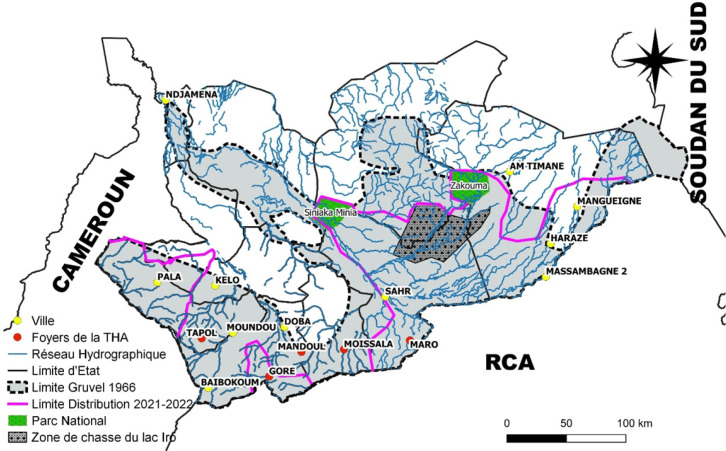
Nouvelle limite nord proposée des glossines au Tchad d'après les résultats 2021-2022 (ligne vio-lette), évolution par rapport à 1966 (tirets noirs), et localisation des foyers de THA (points rouges) The proposed new Northern limit of tsetse in Chad (purple line) in comparison with the old one of 1966 (black dotted line), and geographical location of HAT foci (red dots)

Les résultats des piégeages réalisés lors de cette étude ont été comparés à ceux des travaux de Gruvel [14] et Cuisance [9]. La nouvelle limite nord des tsé-tsé au Tchad proposée, dans la limite de ces observations, est représentée par la ligne en violet sur la carte de la Figure 3. On observe une forte contraction de l'aire de distribution des tsé-tsé du nord vers le sud en comparaison à la limite décrite par Gruvel [14], ainsi que celle de Cuisance [9] avec des superficies importantes perdues par les tsé-tsé. Dans la partie est, la présence de zones de faune sauvage permet encore le maintien de *G. morsitans submorsitans.* Pour toutes ces espèces de tsé-tsé, une fragmentation de leur aire de distribution en « poches » spatialement distinctes et discontinues, est de plus en plus la rèele.

### Évolution de la limite de présence des tsé-tsé au regard des foyers de THA

Au Tchad, la transmission de la THA est assurée par *G. f. fuscipes.* Or, nos résultats (Fig. 3) montrent qu'elle n'est plus retrouvée qu’à Goré et Maro. Elle n'est plus capturée à Tapol ni à Moissala. Elle est bien présente en termes de répartition géographique dans le foyer du Mandoul, mais la lutte antivectorielle qui y est menée depuis 2013 a entraîné sa quasi-disparition [18, 21, 27], qui est donc ici une absence « provoquée ».

## Discussion

Les premières enquêtes entomologiques sur la distribution géographique des glossines dans le sud du Tchad, qui datent de 1966 [14], avaient mis en évidence la présence de 3 espèces de glossines que sont *G. f. fuscipes, G. tachinoides* et *G. morsitans submorsitans.* Ensuite, l’étude de 1996 [9] avait permis non seulement de confirmer la présence de ces 3 taxons de glossines, mais aussi d'observer une première rétraction de leur aire de distribution, du fait des changements globaux (sécheresse et pression anthropique sur le couvert végétal), et de certaines opérations locales de lutte anti-tsé-tsé. On peut par exemple citer la campagne d’éradication réussie de *G. tachinoides* par Robert Tibayrenc en 1972-73 par pulvérisation terrestre d'un organo-chloré dans la vallée du Bas-Chari, qui explique la disparition de cette glossine dans son aire de distribution la plus au nord du Tchad [33]. Dans notre étude, l'identification de *G. morsitans submorsitans, G. f. fuscipes* et G. *tachinoides* corrobore les résultats de Gruvel [14] et de Cuisance [9]. Cette diversité d'espèces peut s'expliquer par la diversité biogéographique du sud du Tchad, qui offre un habitat favorable à chacune de ces espèces.

La plus forte proportion de *G. morsitans submorsitans* capturées, malgré son recul avéré, peut s'expliquer de deux manières. D'une part, la savane boisée constitue le principal biotope de notre zone d’étude, et, d'autre part, des pièges ont été posés dans des espaces protégés de l'est où cette espèce est présente en grand nombre, grâce à de fortes densités de grands mammifères (cob de Buffon, bubale, hippotrague, buffle, phacochère, guib harnaché, etc.) qui constituent leur principale source de nourriture [25]. Ces résultats rejoignent ceux rapportés au Nigeria, ou au Burkina Faso [7, 8, 10, 35], où *G. morsitans submorsitans* est également en recul, restreinte aux zones protégées abritant la faune sauvage [voir également 26].

*G. tachinoides* a été capturée dans les provinces du Mayo-Kebbi, du Moyen-Chari, du Guéra, du Salamat et du Chari-Baguirmi en faibles densités, dans les habitats où les conditions lui sont favorables : présence d'hôtes nourriciers sauvages ou domestiques, végétation bordant lacs, rivières et leurs différents affluents, ainsi que la présence des forêts-galeries le long des cours d'eau.

*G. f. fuscipes* a été capturée dans le Logone Oriental, le Mayo-Kebbi et le Guéra. Lors d’études précédentes [18, 21] *G. f. fuscipes* avait également été capturée dans la province du Logone Occidental et du Moyen-Chari. Ces zones sont les plus humides du pays avec une multitude de cours d'eau permanents (Mandoul, Grande Sido) et des forêts-galeries, qui expliquent la présence de cette espèce susceptible d’être le vecteur de la THA.

Cette étude a permis de définir une nouvelle limite nord de distribution des glossines dans le sud du Tchad. Cette limite nord a glissé vers le sud de manière importante par rapport à celle définie par Gruvel [14], également par rapport aux captures de Cuisance [9], qui a utilisé le même type de méthodologie, par piégeage. Gardons à l'esprit quelques limites de ces études (incluant celle-ci) basées uniquement sur la présence de glossines dans les pièges, puisque leur absence serait beaucoup plus difficile à démontrer que leur présence, et nécessiterait beaucoup plus d'efforts de piégeage ainsi que des outils biostatistiques permettant d'approcher avec certitude cette absence. Nous avons donc conscience qu'un piège n'ayant pas capturé de tsé-tsé ne signifie pas forcément son absence totale, qui peut aussi varier selon les saisons. Toutefois, les entomologistes spécialistes des tsé-tsé ayant une forte connaissance de ces terrains ont souvent pu, par simple observation, se rendre compte de la disparition du couvert végétal des galeries riveraines, et de la faune sauvage sur beaucoup de ces points de piégeage sans tsé-tsé, en rapport avec les piégeages de Gruvel et Cuisance.

Cette réduction observée de l'aire de répartition traduit donc une tendance réelle, qui peut s'expliquer principalement par la forte pression anthropique liée à l'augmentation des densités de populations [6] et par les différentes sécheresses survenues, et leurs conséquences. Parmi ces dernières figurent les déplacements massifs des éleveurs et agriculteurs du Nord vers le Sud à la recherche d'eau, de terres agricoles fertiles et de pâturages, le déboisement de la forêt riveraine et de la végétation interfluve pour l'agriculture, la disparition de la faune sauvage dans les zones non protégées, qui sont autant de facteurs contribuant à la réduction et la disparition des tsé-tsé, comme décrit par ailleurs en Afrique de l'Ouest [8] et par exemple au Burkina Faso [7].

## Conclusion

Les résultats de ce travail confirment la présence de 3 taxons de tsé-tsé, *G. m. submorsitans, G. f. fuscipes* et *G. tachinoides* dans le sud du Tchad. Ils mettent également en évidence une nette rétraction de l'aire de distribution des tsé-tsé au Tchad en comparaison avec les études précédentes, avec un glissement vers le sud de leur limite nord de distribution, ainsi qu'une fragmentation de cette aire en poches discrètes. Ces informations pourront être avantageusement utilisées par les programmes nationaux en charge du contrôle des trypanosomoses humaines et animales, et seront complétées et améliorées dans le cadre du développement d'un atlas national des mouches tsé-tsé et de la TAA en préparation [2].

## Remerciements

Ces activités ont été menées dans le cadre du projet « TRYPA-NO » financé par la Bill and Melinda Gates Foundation (convention N° INV-001785), et du projet « COMBAT » financé par le programme Horizon 2020 de l'Union européenne (convention N° 101000467). Nous remercions les autorités administratives, militaires, traditionnelles et coutumières des zones investiguées pour leurs orientation et protection durant ce travail. Nous remercions le Ministère tchadien de l'environnement, à travers la Direction générale de la faune et de la flore. Nous remercions également les techniciens, les gardes forestiers, les guides et les chauffeurs pour leurs accompagnements lors de ces enquêtes de terrain. La FAO a fourni une assistance technique à cette initiative dans le cadre du Programme de lutte contre la trypanosomose africaine (PLTA - www.fao.org/paat/fr). Nous profitons également de cette opportunité pour rendre un hommage appuyé au Dr Peka Mallaye et au Dr Djabou Guindja, qui ont largement contribué à la réalisation de cette étude; paix à leurs âmes. Enfin nous remercions sincèrement les relecteurs qui nous ont permis d'améliorer le manuscrit.

## Décharge de responsabilité

Les appellations employées dans cet article et la présentation des données qui y figurent n'impliquent de la part de l'Organisation des Nations unies pour l'alimentation et l'agriculture (FAO) aucune prise de position quant au statut juridique ou au stade de développement des pays, territoires, villes ou zones ou de leurs autorités, ni quant au tracé de leurs frontières ou limites.

## Contribution des auteurs

Ce travail a été réalisé en collaboration avec l'ensemble des auteurs.

BGM, FC, PS ont conçu l’étude et ses protocoles. BGM, MA, JD, WY, LS, IB, DS, DH et RK ont tous participé au lourd travail de terrain dans des conditions difficiles. RK et WY ont élaboré les cartes sous la supervision et les recommandations de FC, PS et GC. BGM et DS ont rédigé la première version du manuscrit, qui a été revue par PS et par tous les co-auteurs lors des différentes versions. Tous les auteurs ont lu et approuvé le manuscrit soumis et le manuscrit révisé et final.

## Liens d'intérêts

Les auteurs ne déclarent aucun lien d'intérêts.
